# Mesh shrinkage after transabdominal preperitoneal inguinal hernia repair

**DOI:** 10.1038/s41598-023-31088-8

**Published:** 2023-03-08

**Authors:** Ikuo Watanobe, Shozo Miyano, Michio Machida, Hiroyuki Sugo

**Affiliations:** Department of General Surgery, Juntendo University Nerima Hospital, 3-1-10 Takanodai, Nerima-ku, Tokyo, 177-8521 Japan

**Keywords:** Medical research, Signs and symptoms

## Abstract

Synthetic mesh is now used for inguinal hernia repair in most cases. It is well known that the indwelling mesh contracts after placement in the body, regardless of the material. The aim of this study was to develop a method for indirect measurement of the mesh area postoperatively that allows for easy comparison with the condition of the mesh immediately after surgery. X-ray-impermeable tackers were used to fix the mesh, and changes of the indwelling mesh after surgery were measured indirectly using two mesh materials. This study involved 26 patients who underwent inguinal hernia repair with a polypropylene or polyester mesh (13 patients each). Polypropylene showed a stronger tendency to shrink, but there was no significant difference between the materials. For both materials, some patients showed relatively strong shrinkage and others showed relatively weak shrinkage. The group with the strong shrinkage had significantly higher body mass index. The results of the present study showed that mesh surly shrinked over time and there was no adverse effect of mesh shrinkage on the patients outcomes in this population. Mesh would shrink over time regardless of the sort of mesh but it did not affect the patients outcomes.

## Introduction

**Figure 1:**
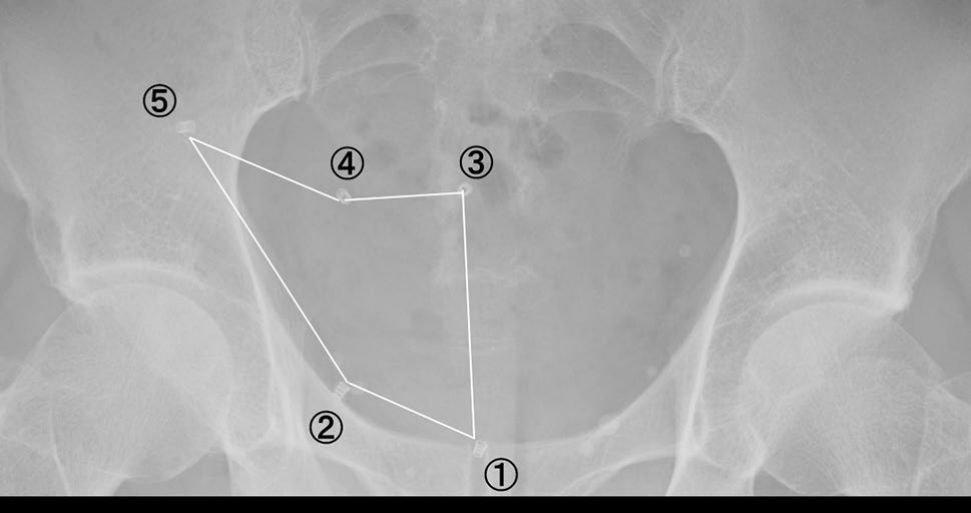
Positions of tackers and area measurement. (1) Dorsal aspect of the pubic bone, (2) Cooper’s ligament (3) back surface of the rectus abdominis muscle, (4) the inner edge of the inferior epigastric artery and vein, and (5) outer upper edge of the mesh. The lines connecting these points were traced, and then the area surrounded by those lines was automatically calculated.

Inguinal hernia repair surgery began with tissue repair methods, such as the McVay method or Bassini method, but now repair often involves radical surgery with abdominal wall reinforcement using synthetic materials. The surgical approach has also changed from an inguinal incision to laparoscopic surgery, such as the transabdominal preperitoneal (TAPP) approach or totally extra-peritoneal approach. As a result, the myopectineal orifice (MPO) area and hernia gate, which are considered the most frequent sites of inguinal hernia, can be directly observed, and the recurrence rate has been greatly reduced by being able to reliably cover the defect with synthetic mesh. However, there are some cases of recurrence due to postoperative changes such as mesh shrinkage or migration. Here, we report mesh areas indirectly measured using X-ray-impermeable tackers for two mesh materials—polypropylene (PP : pore size 1.67 × 1.67 mm, weight 40.3 g/m^2^) and polyester (PE : pore size 1.7 × 1.3 mm, weight 117 g/m^2^)—and changes of the mesh after inguinal hernia repair (Fig. 1).

## Results

All 26 patients were male, with a mean age of 75 (range 65–91) years. Body mass index (BMI) was 22.3 (17.3–28.4). The affected side was the right in 15 cases and the left in 8 cases. The mean operation time was 52 min, and intraoperative blood loss was < 2 g in all cases. We used PP mesh in 13 cases and PE mesh in 13 cases.

**Figure 2:**
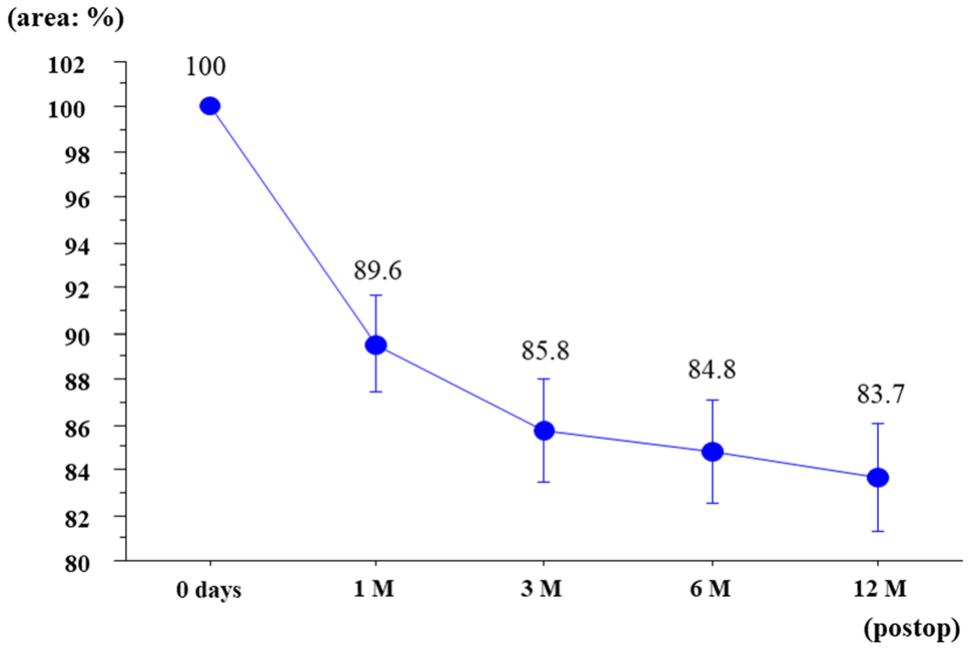
Postoperative changes in mesh area over time (the area immediately after surgery was taken as 100%).

**Figure 3:**
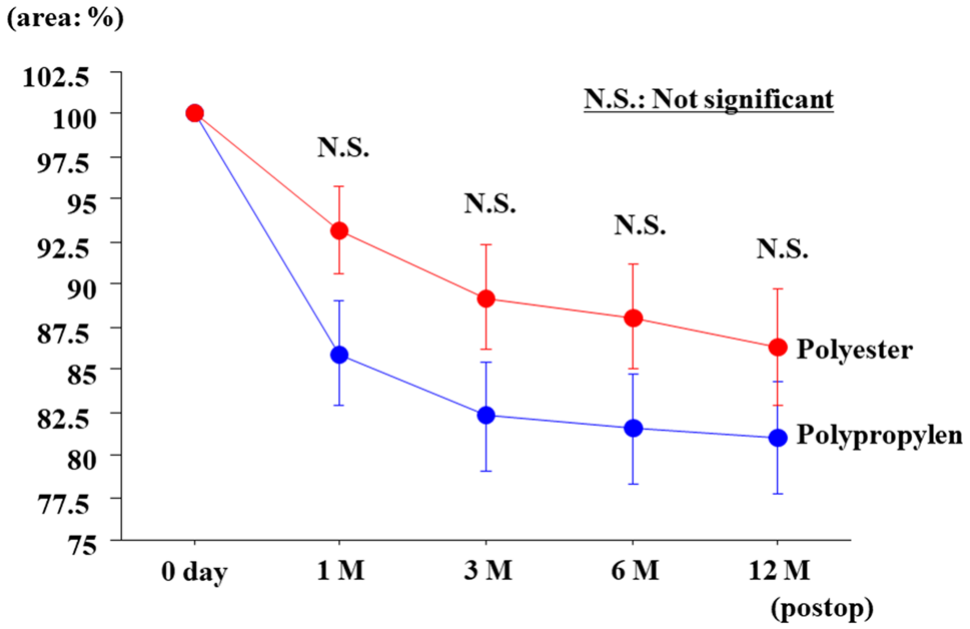
Comparison of polyester meshes and polypropylene meshes. Both types of meshes showed similar changes in area due to shrinkage. Polypropylene meshes tended to shrink slightly more than polyester meshes.

The change ratio in the calculated area was 89.6% at 1 month after surgery, 85.8% at 3 months after surgery, 84.8% at 6 months after surgery, 83.7% at 12 months after surgery (Fig. 2). Mesh shrinkage progressed marked in the first month and then more gradually until 12 months after surgery. Mesh shrinkage tended to be larger for PP compared with PE, but the difference was not significant (Fig. 3). In terms of complications, 2 patients developed visible seromas, but no puncture was required and the seromas had disappeared by the examination 6 months after surgery. Chronic postoperative inguinal pain (CPIP), recurrence of inguinal hernia, and any other postoperative complications were not recognized in this study.

**Figure 4:**
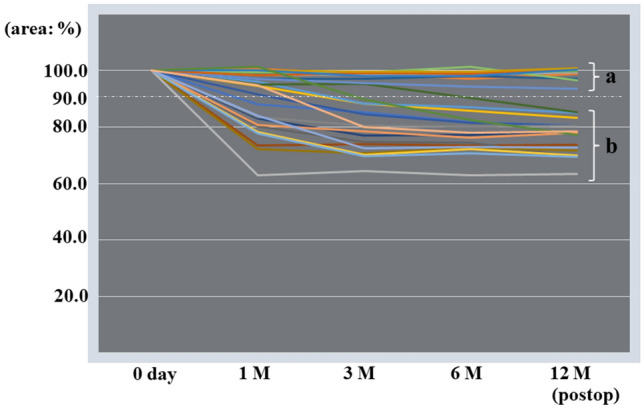
Changes in mesh area in all patients. Patients were divided into a group with little shrinkage (**a**) shrinkage to ≥ 90%) and a group with more shrinkage (**b**) shrinkage to < 90%) throughout the 12-month observation period.

Based on the change ratio of the mesh area, we divided them into a group with shrinkage to ≥ 90% and a group with shrinkage to < 90% throughout the observation period (Fig. 4). Comparing these two groups, we found that BMI was significantly higher in the group with shrinkage to < 90% (Table 1).

**Table 1:** Patient background characteristics.

Mesh size (12 months postop)		≥ 90%	< 90%
Age (y)	N.S	76.2 (65–91)	74.5 (66–85)
**BMI**	***p* < 0.05**	**20.8 (17.3–27.4)**	**23.2 (18.7–28.4)**
Operation time (min)	N.S	73.0 (55–93)	72.7 (58–87)
Blood loss (g)	N.S	1.3 (1–3)	1.2 (1–3)
Hernia gate diameter (mm)	N.S	27 (24–33)	29 (23–35)

Bold indicates a statistically significant difference. BMI, body mass index.

**Figure 5:**
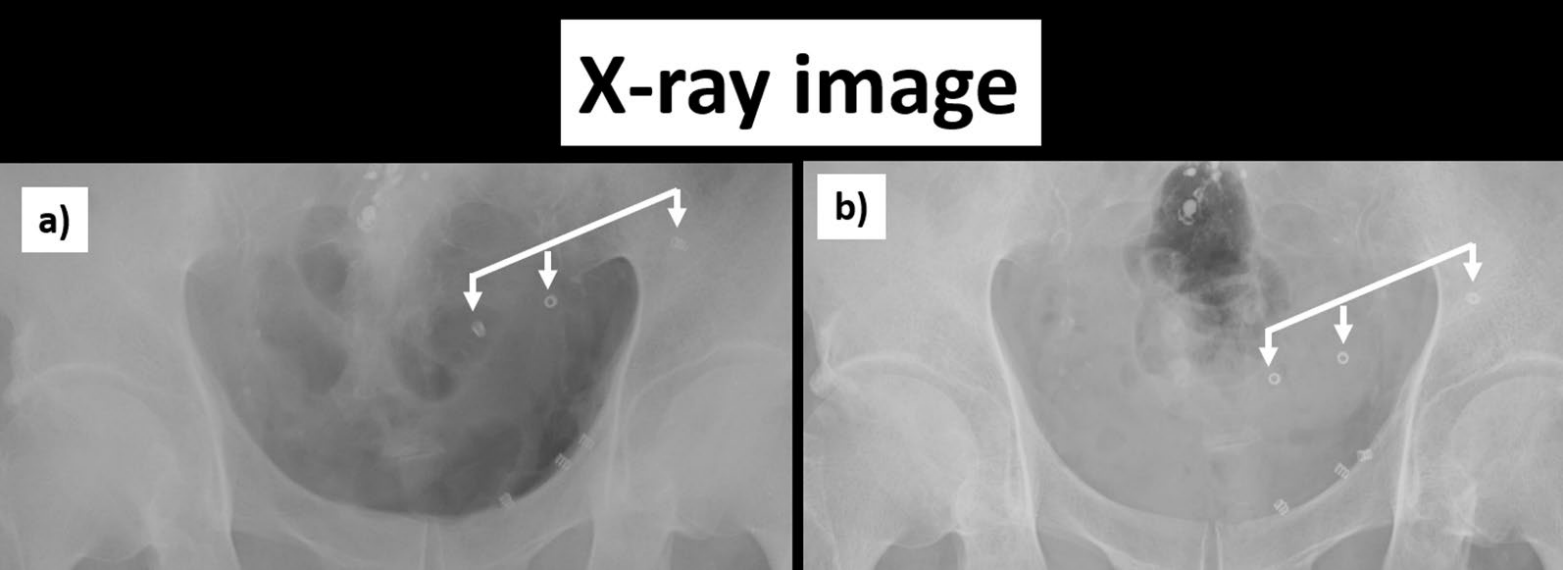
Abdominal x-rays after surgery (**a**) immediately after surgery; (**b**) 1 month after surgery) for the case with the greatest mesh shrinkage. The positions of 3 tackers placed on the ventral side (back surface of the rectus abdominal muscle, inner edge of the inferior epigastric artery and vein, and outer upper edge of the mesh) moved toward the dorsal aspect of the pubic bone and Cooper’s ligament, which were in the deepest part of the surgical field. The positional relationships of these 3 tackers were maintained as they moved.

We also found that in all 26 cases, the tackers fixed to dorsal aspect of the pubic bone and Cooper’s ligament did not move at all. In patient whose mesh had shrunken, the positions of the tackers fixed to the back surface of the rectus abdominal muscle, inner edge of the inferior epigastric artery and vein, and outer upper edge of the mesh moved toward the dorsal aspect of the pubic bone and Cooper’s ligament along with mesh shrinkage (Fig. 5). At four annual postoperative follow-up visits, groin pain was mostly relieved within one month after surgery. In addition, no patient showed a decline in QOL during this observation period.

## Discussion

The postoperative recurrence rate of inguinal hernia has recently been greatly reduced by replacing tissue repair methods with mesh repair methods^1,2^. However, there are still exists some cases of recurrence, and intraoperative findings in recurrent cases have revealed mesh shrinkage and migration. Some studies have investigated in vivo mesh behavior using special mesh examined by computed tomography^3^ or magnetic resonance imaging (MRI)^4^. However, many of those studied involved ventral hernia or animal models. Few studies have investigated mesh behavior in humans after inguinal hernia repair^5,6^.

A detailed study in animal models found that tissue cells penetrate well into the mesh early after surgery, which is important to fix the mesh and to prevent mesh shrinkage or migration^7^. It is important to fix the mesh to the abdominal wall in order to prevent mesh shrinkage or migration early after surgery before tissue development is completed. Shrinkage of mesh integrated with surrounding tissue following the postoperative acute phase depends on subsequent inflammation and the accompanying scar formation^8^. In other words, the ideal mesh has good penetration of tissue cells into the mesh in the early phase after surgery, and it can be considered ideal for a mesh material to cause less inflammation.

The degree of mesh shrinkage in vivo varies among reports^9,10^. Although the mesh shrinkage has been reported to progress greatly and reaches a plateau of gradual progression or contraction within 1 month after surgery, this was attributable to differences in the mesh material, the presence or absence of suture fixation, and whether the site of the indwelling mesh was the abdominal wall or pelvic floor. Although our study involved an indirect assessment, we evaluated the mesh behavior after TAPP surgery in humans. Furthermore, only abdominal X-rays were required for evaluation, and costly examinations such as MRI used in previous studies of mesh behavior were not necessary. Evaluation could be performed during routine postoperative follow-up.

In this study, mesh shrinkage showed the strongest contraction from immediately after surgery to 1 month later. Consistent with previous reports, this result seems to reflect that most shrinkage occurs before the mesh integrates with the surrounding tissue. Muysoms et al. reported that the strongest change in mesh shrinkage occurred during the first month after surgery, but that study did not compare the mesh area with evaluation under the same conditions immediately after surgery and 1 month later^5^. An advantage of our study is that measurements were performed under the same conditions at all five time points from immediately after surgery to 12 months later. This was possible because of the ease of evaluation using abdominal radiography. However, immediately after surgery, although degassing was performed to remove intra-abdominal gas at the end of the operation, there was possibly a slight influence of residual intra-abdominal gas. However, this effect was thought to be minimal because the tacker was fixed to the pelvic floor with the patient supine, so any residual gas while taking the x-ray would mostly move to the center of abdominal, away from the pelvic floor.

We found no significant difference in shrinkage between PE and PP, though the shrinkage tended to be stronger in PP materials. This result was not further studied histopathologically in this study, but it seems to reflect the difference between these materials in the penetration of tissue cells into mesh, consistent with a report by Gonzalez et al.^11^.

Regardless of mesh material, the mesh shrinkage over time could be seen in all 26 cases, and the patients were divided into two groups showing a tendency for relatively little shrinkage (to ≥ 90%) or relatively stronger shrinkage (to < 90%) at 12 months after surgery. When we compared background characteristics between the groups, we found a significant difference in only BMI (p > 0.05). Also, in the group with shrinkage to < 90%, there was a tendency for shrinkage to continue beyond 1 month after surgery. This fact indicates that shrinkage continues after the acute phase has passed and the mesh is integrated into the tissue. A possible explanation for this is that patients with high BMI was likely have an adipose tissue component in the abdominal wall, and the adipose tissue can cause the whole tissue containing the mesh to slip. This can be inferred from the fact that there was no change in the tackers fixed to the strong supporting tissue of the pubic bone and Cooper’s ligament. Figure 5 is a radiograph that shows the change of tacker positions at 1 month after surgery in the patient with the largest mesh shrinkage. In this patient, 2 tackers were fixed to Copper’s ligament. The positions of 3 tackers placed on the ventral side (i.e., those fixed to the back surface of the muscle, inner edge of the inferior epigastric artery and vein, and outer upper edge of the mesh) moved toward the tackers fixed to the pubic bone and Cooper’s ligament, which were in the deepest part of the surgical field. As seen in these radiographs, the positional relationships of the 3 tackers on the ventral side were maintained after they moved, suggesting that the mesh was not damaged and retained the tackers. Change of tacker positions associated with mesh shrinkage was a phenomenon observed on radiographs of all 26 patients at all time points. Taken together, this study confirmed that, in mesh fixation using nonabsorbable tackers, the tackers were firmly fixed to the solid structures (i.e., dorsal aspect of the pubic bone and Cooper’s ligament), while those fixed to the structures such as peritoneal tissue, fascial tissue, and adipose tissue moved along with mesh shrinkage. Because we cannot evaluate mesh condition using radiolucent fixation devices repair, the use of non-radiolucent fixation devices in obese patients with a high BMI would provide useful information for evaluating the mesh condition and planning the next treatment when pain or symptoms of recurrence appear at a later date, simply by taking a pelvic x-ray.

In previous reports on animal models, detailed examinations have been conducted, such as histopathological changes and measurement of the fixation force to actual tissues, but it has been difficult to examine the progression of long-term changes. Up to now, studies in humans have involved expensive methods such as a special mesh and MRI examination, which cannot be easily performed as part of routine postoperative follow-up. In this series, we investigated changes of mesh over 12 months following TAPP surgery on x-rays. In daily medical practice, the condition of the mesh after hernia surgery is unfortunately only seen after major complications such as recurrence, mesh infection, or chronic pain requiring reoperation, or if the patient has another disease that requires surgical treatment. In particular, cases of recurrence and CPIP are often associated with a high degree of mesh shrinkage and migration. It is significant that a two-dimensional pelvic x-ray, rather than an expensive and invasive CT or MRI scan, provides useful mesh conditional information for the next stage of treatment. Our present study was small in scale for a comparative study and limited in the type of mesh. Furthermore, there is the drawback of using two-dimensional images to evaluate three-dimensional structures. But we expect that our method will be useful, particularly for examination when we need to determine the state of the mesh postoperatively at low cost.

## Methods

Participants were patients aged ≥ 20 years who were diagnosed with inguinal hernia at our hospital between June 2019 and December 2021. Inclusion criteria were the lateral or medial type of unilateral inguinal hernia according to the European Hernia Society classification. In total, 28 patients were enrolled who had no history of lower abdominal surgery or TAPP repair and consented to participate in the study. The operation was performed by a single surgeon who was certified under the technical certification system for laparoscopic surgery in Japan. Two of the 28 cases who were found to have bilateral inguinal hernia during the operation were excluded. Patients with an American Society of Anesthesiologists physical status ≥ Class 3, incarcerated hernia, or recurrent hernia, those who were pregnant, or those judged to have difficulty in attending follow-up hospital visits after surgery were excluded.

We used a PP or PE mesh, and surgical stainless steel tackers. All surgeries used mesh of sufficient size to cover the MPO area, and tackers were fixed in five places: dorsal aspect of the pubic bone, Cooper’s ligament, the back surface of the rectus abdominis muscle, the inner edge of the inferior epigastric artery and vein, and the outer upper edge of the mesh. Since the introduction of TAPP repair in 2013 at our hospital, we have performed tacking on firm soft tissues such as bone and muscle instead of fatty tissues in over 800 cases to prevent early postoperative mesh migration and turn over. To avoid CPIP, tacking is not performed in the so-called danger zone (the lateral dorsal area from the spermatic duct to just above the ilio-pubic tract). The peritoneum was continuously sutured and closed with monofilament absorbent sutures in all cases.

We measured the mesh area surrounded by tackers on abdominal x-ray images at 5 time points: immediately after surgery, 1 month after surgery, 3 months after surgery, 6 months after surgery, and 12 months after surgery. The mesh area measurement was performed using Fujitsu Lifecare Solution HOPE Life-Mark-PACS version 1.28.00 on the monitor screen (Fig. 1). Since the three-dimensional pelvic floor structure is projected onto a plane and measured, the calculation result for the area was not directly used, and mesh shrinkage was evaluated at the ratio of change in area over time. Immediately after the operation, an abdominal x-ray was taken in a supine position in the operating room, after sufficient degassing was performed to avoid the influence of residual gas in the intra-abdominal space. X-rays at the subsequent time points were also taken in the supine position so as to have the same conditions. In each photograph, close attention was paid so that the pelvis was facing the front.

In the mesh area measurement, we assumed that deviation of the tackers was misalignment of mesh. In other words, we assumed there was no corruption of mesh or no deviation of the tackers from the mesh. To minimize the measurement error, the area measurements were performed five times by an author (S.M.), and the average value was used as the area. The calculated area immediately after surgery was taken as 100% and the change ratio of the measured area was determined after 1 month, 3 months, 6 months, and 12 months. The Mann–Whitney U test was used to test for significant differences (significant : *p* < 0.05). In addition, at four postoperative visits per year, the VAS (visual analog scale) score was used to assess groin pain, and a questionnaire was administered regarding health and quality of life changes.

### Ethical approval and informed consent

This study received institutional review board approval, and it conforms to the provisions of the Declaration of Helsinki. This study received institutional review board approval by Research Ethics Committee, Faculty of Medicine, Juntendo University. The subject gave informed consent, and patient anonymity was preserved.

## Data Availability

The datasets generated and/or analyzed during the current study are available from the corresponding author on reasonable request.
